# Characteristics of Medical Cannabis Patients and Clinicians in 7 US States

**DOI:** 10.1001/jamanetworkopen.2025.6925

**Published:** 2025-04-24

**Authors:** Kevin F. Boehnke, Rachel Sinclair, Felicia Gordon, Tristin Smith, Douglas R. Roehler

**Affiliations:** 1 Anesthesiology Department, Chronic Pain and Fatigue Research Center, University of Michigan Medical School, Ann Arbor; 2 Michigan Psychedelic Center, University of Michigan Medical School, Ann Arbor; 3 Cannabis Strategy Unit, Division of Overdose Prevention, National Center for Injury Prevention and Control, Centers for Disease Control and Prevention, Atlanta, Georgia; 4 Oak Ridge Institute for Science and Education, Oak Ridge, Tennessee

## Abstract

This cross-sectional study investigates county-level sociodemographic characteristics of patients who use medical cannabis and their authorizing physicians in 7 US states.

## Introduction

In the US, medical cannabis (MC) is legal in 39 states and the District of Columbia, with over 4.1 million registered US MC patients and 29 500 clinicians authorizing MC licensure as of December 2022.^1^ However, little is known about county-level factors associated with rates of MC patient licensure and MC authorizing clinicians. Drawing from a social determinants of health framework,^2^ we investigated county-level sociodemographic characteristics associated with MC licensure and authorization in 7 US states.

## Methods

In this cross-sectional study, we analyzed county-level characteristics from publicly available state reports and data requests from the 7 states (eTable in Supplement 1) reporting both registered MC patients and authorizing clinicians in 2022. Collection methods are reported elsewhere.^1^ Since available data were deidentified, ethics review and informed consent were not conducted, in accordance with 45 CFR §46. We followed the STROBE reporting guidelines for cross-sectional studies. We collected county-level demographic characteristics, including male-to-female ratio, median age, percentage from minoritized racial and ethnic groups (American Indian or Alaska Native, Asian, Black or African American, Native Hawaiian or Other Pacific Islander, and any other race or ethnicity not otherwise specified), median household income, unemployment percentage, uninsured percentage, poverty percentage, disability percentage, and veteran percentage via the American Community Survey.^3^ We included measures of social vulnerability using the Social Vulnerability Index (SVI)^4^ and classified counties as metropolitan or nonmetropolitan using Rural-Urban Continuum Codes.^5^ We calculated MC patient and authorizing clinician county-level rates per 10 000 residents using county-level populations from the 2022 US census. We assessed associations between patient and authorizing clinician rates with county-level characteristics and state legality of nonmedical adult cannabis use as of 2022 using negative binomial regression. Our final models used bidirectional stepwise regression (2-sided tests, α = .05) and clustered SEs by state to limit the influence of states with large numbers of counties, with selection based on Akaike information criterion values. Analyses were performed in R statistical software version 4.1.1 (R Project for Statistical Computing).

## Results

Our analyses included 262 counties (103 metropolitan and 159 nonmetropolitan), with mean (SD) age 42.2 (5.1) years and mean (SD) percentage of individuals from minoritized racial and ethnic groups 7.93% (9.76%). There were 75.5 MC patients per 10 000 population and 1.4 authorizing clinicians per 10 000 population (Table 1). Stepwise multivariate negative binomial regression analyses of patient rate demonstrated negative associations with higher percentage of individuals from minoritized racial and ethnic groups (B, 0.979; 95% CI, 0.960-0.999; *P* = .04) and unemployment percentage (B, 0.928; 95% CI, 0.863-0.999; *P* = .048), but positive associations with county-level median household income (B, 1.102; 95% CI, 1.057-1.148; *P* < .001) and uninsured percentage (B, 1.165; 95% CI, 1.056-1.285; *P* = .002) (Table 2). For clinician rate, there were negative associations with male-to-female ratio (B, 0.944; 95% CI, 0.906-0.983; *P* = .005) and unemployment percentage (B, 0.733; 95% CI, 0.666-0.806; *P* < .001) and positive associations with county-level median household income (B, 1.325; 95% CI, 1.052-1.665; *P* = .02), veteran percentage (B, 1.180; 95% CI, 1.060-1.315; *P* = .003), and SVI (B, 1.149; 95% CI, 1.031-1.279; *P* = .01) (Table 2).

**Table 1.** Descriptive Statistics of 262 Counties in 7 States Included in Analysis, 2022^a^

Variable	Value, mean (SD)
Ratio of male to female individuals^b^	102.1 (6.45)
Age, y	42.19 (5.05)
Population from minoritized racial and ethnic groups, %^c^	7.93 (9.76)
County-level median household income, $	68 810.66 (16 990.90)
Unemployment rate, %	4.90 (2.27)
Uninsured rate, %	6.14 (2.64)
Poverty rate, %	8.24 (3.94)
Disability rate, %	15.21 (4.55)
Veteran rate, %	7.53 (1.91)
Patient rate per 10 000 population, median (IQR)	75.47 (50.54)
Clinician rate per 10 000 population, median (IQR)	1.38 (2.50)

^a^ Metropolitan vs nonmetropolitan breakdown of 7 included states are Delaware (2 metropolitan, 1 nonmetropolitan), Maine (5 metropolitan, 11 nonmetropolitan), Minnesota (27 metropolitan, 60 nonmetropolitan), New Hampshire (3 metropolitan, 7 nonmetropolitan), New York (37 metropolitan, 25 nonmetropolitan), Utah (9 metropolitan, 20 nonmetropolitan), and West Virginia (20 metropolitan, 35 nonmetropolitan).

^b^ County-level sex ratio is defined as the number of male individuals per 100 female individuals.

^c^ Includes census categories of American Indian or Alaska Native, Asian, Black or African American, Native Hawaiian or Other Pacific Islander, and any other race or ethnicity not otherwise specified.

**Table 2.** Stepwise Regression Modeling for Final Models of Patient Rate and Clinician Rate Using Negative Binomial Regression and Bidirectional Stepwise Regression in 7 US States^a^

Variable	Patient rate^b^		Clinician rate^b^	
	B (95% CI)	*P* value	B (95% CI)	*P* value
Ratio of male to female individuals	0.989 (0.964-1.015)	.40	0.944 (0.906-0.983)	.005
Median age	NA	NA	NA	NA
Percentage of population from minoritized racial and ethnic groups^c^	0.979 (0.960-0.999)	.04	NA	NA
Metropolitan status	NA	NA	0.727 (0.485-1.090)	.12
County-level median household income (scaled per $10 000)	1.102 (1.057-1.148)	<.001	1.325 (1.052-1.665)	.02
Unemployment rate	0.928 (0.863-0.999)	.048	0.733 (0.666-0.806)	<.001
Uninsured rate	1.165 (1.056-1.285)	.002	NA	NA
Poverty rate	NA	NA	NA	NA
Disability rate	NA	NA	NA	NA
Veteran rate	1.087 (0.940-1.257)	.26	1.180 (1.060-1.315)	.003
Nonmedical adult cannabis use legal status	2.049 (0.745-5.637)	.16	NA	NA
Social Vulnerability Index (scaled per 0.1 unit change)^d^	NA	NA	1.149 (1.031-1.279)	.01

Abbreviation: NA, not applicable.

^a^ The 7 included states are Delaware, Maine, Minnesota, New Hampshire, New York, Utah, and West Virginia.

^b^ Patient rate is the number of medical cannabis–using patients per 10 000 census population. The clinician rate is number of authorizing clinicians per 10 000 census population. All results are produced using negative binomial regression. Models were selected using a bidirectional stepwise regression approach with selection based on Akaike information criterion values. SEs were clustered by state. Each variable listed in the table was considered in the stepwise modeling approach. Variables with coefficients were in the final model after first considering all covariates. *P* values were adjusted using Bonferroni correction.

^c^ Includes census categories of American Indian or Alaska Native, Asian, Black or African American, Native Hawaiian or Other Pacific Islander, and any other race or ethnicity not otherwise specified.

^d^ The Social Vulnerability Index ranges from 0 to 1. For inclusion in this mode, the Social Vulnerability Index was scaled per 0.1 unit change.

## Discussion

In this cross-sectional study, we identified several factors associated with MC patient and authorizing clinician rates in 7 US states. Some factors aligned between patient and clinician models, such as higher county-level household income and lower unemployment. However, future research is needed to understand discrepancies between models, such as associations between uninsurance percentage and MC patient rate as well as between SVI and authorizing clinician rate. The association between veteran population and higher rates of authorizing clinicians may reflect veteran efforts to expand MC access and higher prevalence of qualifying conditions for MC licensure among veterans (eg, chronic pain or posttraumatic stress disorder).^6^ Limitations include that states publishing county-level data may not represent other states with MC legality, we measured clinician rate rather than number of authorizations per clinician (data that are currently unavailable), and other unmeasured confounders that may influence our outcomes. As cannabis policy evolves,^1^ it is important to continue future research efforts focused on understanding factors associated with MC patient and clinician rates. Doing so will help more accurately target appropriate substance use resources and education related to cannabis in communities with the most need.

## Notes

Supplement 1. **eTable.** Characteristics of 7 states included in analysis, 2022

Supplement 2.
Data Sharing Statement
