# Supplementary material for: Characteristics of Medical Cannabis Patients and Clinicians in 7 US States
Source: JAMA Netw Open. 2025 Apr 24;8(4):e256925. doi: 10.1001/jamanetworkopen.2025.6925 (PMC12022805; doi:10.1001/jamanetworkopen.2025.6925)
Supplement: Supplement 1. — eTable. Characteristics of 7 states included in analysis, 2022 [file jamanetwopen-e256925-s001.pdf]

Supplemental Online Content

Boehnke KF, Sinclair R, Gordon F, Smith T, Roehler DR. Characteristics of medical cannabis patients and clinicians in 7 US states. *JAMA Netw Open*. 2025;8(4):e256925. doi:10.1001/jamanetworkopen.2025.6925

eTable. Characteristics of 7 states included in analysis, 2022

This supplemental material has been provided by the authors to give readers additional information about their work.

© 2025 Boehnke KF et al. *JAMA Network Open*.

eTable. Characteristics of 7 states included in analysis, 2022

	Delaware	Maine	Minnesota	New Hampshire	New York	Utah	West Virginia
**County Type**							
Metro	2 (66.7%)	5 (31.3%)	27 (31.0%)	3 (30.0%)	37 (59.7%)	9 (31.0%)	20 (36.4%)
Non-Metro	1 (33.3%)	11 (68.8%)	60 (69.0%)	7 (70.0%)	25 (40.3%)	20 (69.0%)	35 (63.6%)
**US Census Region Division**	South Atlantic	New England	West North Central	New England	Middle Atlantic	Mountain	South Atlantic
**Adult Use Legalization Year**	-	2016	-	-	2021	-	-
**Adult use dispensaries open**	-	2020	-	-	2022	-	-
**Medical Use Legalization Year**	2011	1999	2014	2013	2014	2018	2017
**Medical Dispensaries opened**	2015	2011	2015	2016	2016	2020	2021
**Qualifying Requirements**							
Patients	Qualifying condition or symptom listed under state statutes	Qualifying condition or symptom listed under state statutes	Qualifying condition or symptom listed under state statutes	Qualifying condition or symptom listed under state statutes	Qualifying condition or symptom listed under state statutes	Qualifying condition or symptom listed under state statutes	Qualifying condition or symptom listed under state statutes
Authorizing Clinicians	MD, DO, PA, APRN Source:	MD, DO, nurse practitioners, PA	MD, DO, PA, APRN	MD, DO, PA, APRN	physicians, nurse practitioners, physician assistants, dentists, podiatrists, and midwives. Complete coursework and can lawfully prescribe controlled substances Complete 2 hour course on cannabis	MD, DO, podiatrists, PA, APRN	Physicians who register with health bureau, complete four-hour course on cannabis, and provide continuing care for patients

Note: State-level characteristics reported as n (%) unless otherwise noted.

© 2025 Boehnke KF et al. *JAMA Network Open*.
