# Supplementary material for: Characteristics of Medical Cannabis Patients and Clinicians in 7 US States
Source: JAMA Netw Open. 2025 Apr 24;8(4):e256925. doi: 10.1001/jamanetworkopen.2025.6925 (PMC12022805; doi:10.1001/jamanetworkopen.2025.6925)
Supplement: Supplement 2. — Data Sharing Statement [file jamanetwopen-e256925-s002.pdf]

Data Sharing Statement

Boehnke. Characteristics of Medical Cannabis Patients and Clinicians in 7 US States. *JAMA Netw Open*. Published April 24, 2025. doi:10.1001/jamanetworkopen.2025.6925

Data
Data available: Yes
Data types: Data (not involving human participants)
How to access data: The data will be available from the authors upon reasonable request (kboehnke@med.umich.edu)
When available: With publication

Supporting Documents
Document types: None

Additional Information
Who can access the data: researchers whose proposed use of the data has been approved
Types of analyses: specified purpose
Mechanisms of data availability: with investigator support after a signed data access agreement
